# Identification of the Neuroinvasive Pathogen Host Target, LamR, as an Endothelial Receptor for the Treponema pallidum Adhesin Tp0751

**DOI:** 10.1128/mSphere.00195-20

**Published:** 2020-04-01

**Authors:** Karen V. Lithgow, Brigette Church, Alloysius Gomez, Emily Tsao, Simon Houston, Leigh Anne Swayne, Caroline E. Cameron

**Affiliations:** a Department of Biochemistry and Microbiology, University of Victoria, Victoria, British Columbia, Canada; b Division of Medical Sciences, University of Victoria, Victoria, British Columbia, Canada; c Department of Medicine, Division of Allergy and Infectious Diseases, University of Washington, Seattle, Washington, USA; University of Kentucky

**Keywords:** adhesin, endothelial cells, invasive bacteria, laminin receptor, syphilis

## Abstract

Treponema pallidum subsp. *pallidum* is the causative agent of syphilis, a human-specific sexually transmitted infection that causes a multistage disease with diverse clinical manifestations. Treponema pallidum undergoes rapid vascular dissemination to penetrate tissue, placental, and blood-brain barriers and gain access to distant tissue sites. The rapidity and extent of T. pallidum dissemination are well documented, but the molecular mechanisms have yet to be fully elucidated. One protein that has been shown to play a role in treponemal dissemination is Tp0751, a T. pallidum adhesin that interacts with host components found within the vasculature and mediates bacterial adherence to endothelial cells under shear flow conditions. In this study, we further explore the molecular interactions of Tp0751-mediated adhesion to the vascular endothelium. We demonstrate that recombinant Tp0751 adheres to human endothelial cells of macrovascular and microvascular origin, including a cerebral brain microvascular endothelial cell line. Adhesion assays using recombinant Tp0751 N-terminal truncations reveal that endothelial binding is localized to the lipocalin fold-containing domain of the protein. We also confirm this interaction using live T. pallidum and show that spirochete attachment to endothelial monolayers is disrupted by Tp0751-specific antiserum. Further, we identify the 67-kDa laminin receptor (LamR) as an endothelial receptor for Tp0751 using affinity chromatography, coimmunoprecipitation, and plate-based binding methodologies. Notably, LamR has been identified as a receptor for adhesion of other neurotropic invasive bacterial pathogens to brain endothelial cells, including Neisseria meningitidis, Haemophilus influenzae, and Streptococcus pneumoniae, suggesting the existence of a common mechanism for extravasation of invasive extracellular bacterial pathogens.

**IMPORTANCE** Syphilis is a sexually transmitted infection caused by the spirochete bacterium Treponema pallidum subsp. *pallidum.* The continued incidence of syphilis demonstrates that screening and treatment strategies are not sufficient to curb this infectious disease, and there is currently no vaccine available. Herein we demonstrate that the T. pallidum adhesin Tp0751 interacts with endothelial cells that line the lumen of human blood vessels through the 67-kDa laminin receptor (LamR). Importantly, LamR is also a receptor for meningitis-causing neuroinvasive bacterial pathogens such as Neisseria meningitidis, Haemophilus influenzae, and Streptococcus pneumoniae. Our findings enhance understanding of the Tp0751 adhesin and present the intriguing possibility that the molecular events of Tp0751-mediated treponemal dissemination may mimic the endothelial interaction strategies of other invasive pathogens.

## Abstract (precis)

Syphilis is a sexually transmitted infection caused by the spirochete bacterium Treponema pallidum subsp. *pallidum.* The continued incidence of syphilis demonstrates that screening and treatment strategies are not sufficient to curb this infectious disease, and there is currently no vaccine available. Herein we demonstrate that the T. pallidum adhesin Tp0751 interacts with endothelial cells that line the lumen of human blood vessels through the 67-kDa laminin receptor (LamR). Importantly, LamR is also a receptor for meningitis-causing neuroinvasive bacterial pathogens such as Neisseria meningitidis, Haemophilus influenzae, and Streptococcus pneumoniae. Our findings enhance understanding of the Tp0751 adhesin and present the intriguing possibility that the molecular events of Tp0751-mediated treponemal dissemination may mimic the endothelial interaction strategies of other invasive pathogens.

## INTRODUCTION

Syphilis is a chronic sexually transmitted infection caused by the spirochete Treponema pallidum subsp. *pallidum* that presents as a multistage disease with diverse clinical manifestations. Despite continued sensitivity to penicillin, syphilis remains a global health concern for which no vaccine is available (1). The World Health Organization (WHO) estimates there are 18 million cases worldwide, with 5.6 million new cases each year (2). Although syphilis rates are highest in low- and middle-income regions (3), higher-income settings, including Canada (4), Europe (5, 6), United Kingdom (7, 8), China (2, 9–11), and the United States (12, 13) are experiencing increasing syphilis rates. Of utmost concern is the increasing occurrence of congenital syphilis, which causes adverse pregnancy outcomes, including stillbirth, early neonatal death, and preterm birth (14). In 2016, the WHO estimated that globally more than 980,000 pregnant women had an active syphilis infection, resulting in an estimated total number of 661,000 congenital syphilis cases (15). Additionally, active syphilis infection increases the risk of HIV acquisition and transmission by three- to fivefold (16–19) and has been associated with increased HIV loads (20, 21). The continued incidence of syphilis worldwide (2, 3) indicates that the current screening and treatment initiatives are insufficient for controlling spread of the disease. An improved understanding of syphilis disease progression and T. pallidum pathogenicity is essential to finding better strategies for syphilis prevention.

Treponema pallidum dissemination is central to the disease progression of syphilis. Following acquisition, organisms rapidly gain entry to the bloodstream, facilitating extensive invasion into distant tissue and organ sites (22–24). The invasive capability of T. pallidum is exemplified by the widespread disease manifestations associated with syphilis, including the development of disseminated lesions and generalized lymphadenopathy during secondary syphilis (25–28), and the gummatous lesions, cardiovascular complications (29–31), and bone destruction that can occur during the tertiary stage of the disease (32–34). Further, invasion into immune privileged areas (35), vertical transmission to the developing fetus (36, 37), and entry into the central nervous system (CNS) can occur early during infection, as illustrated by the finding that up to 40% of individuals with early stage syphilis exhibit CNS involvement (38, 39). While the timing and extent of T. pallidum dissemination are well documented, the molecular mechanisms underlying this process have not been fully elucidated, largely owing to the difficulty associated with studying this organism. These challenges include the historical inability to continuously culture T. pallidum *in vitro* (40), genetic intractability of the organism, inherent fragility of the T. pallidum outer membrane, and lack of conventional virulence factors (34, 41).

Prior studies have identified Tp0751 as a host binding adhesin contributing to T. pallidum dissemination through its interactions with extracellular matrix (ECM) components such as laminin (42–45), fibronectin (44, 45), and fibrinogen (44, 46). Tp0751 has also been characterized as a metalloprotease that can degrade human fibrinogen and laminin (46, 47). Structural determination of Tp0751 revealed a lipocalin fold with an extended N-terminal alpha helix. Unique from lipocalins, Tp0751 lacks the conventional hydrophobic pocket that would normally coordinate binding of lipids or other hydrophobic molecules. The solved Tp0751 structure also lacks both a canonical protease active site and a metal coordination site (45), an unexpected finding considering the proteolytic activity previously described for this protein (46, 47). This structure-function discrepancy may stem from the solving of the structure using a non-wild-type form of Tp0751 (45). However, the true reason for the discrepancy is currently unknown, and since the proteolytic and adhesive activities of Tp0751 have been shown to function independently (47), we focused subsequent investigations on characterizing the adhesive functions of Tp0751. These studies have shown that heterologous expression of Tp0751 in a noninfectious Borrelia burgdorferi model system confers a gain-of-function phenotype for endothelial attachment *in vitro* and *in vivo* (45, 48). Further, biochemical investigations with Tp0751 peptides demonstrated that one face of the lipocalin fold and N-terminal helix confer binding to ECM components, whereas competitive inhibition studies with Tp0751-expressing B. burgdorferi demonstrated that binding to endothelial cells was confined to a discrete region within the lipocalin fold (45). A role for Tp0751 in host attachment is further supported by the finding that Tp0751 immunization partially inhibits bacterial dissemination to distant organ sites upon infectious T. pallidum challenge in an animal infection model (49). Evidence supporting the expression of Tp0751 on the host-interacting, surface-exposed outer membrane of T. pallidum includes the observations that Tp0751 is a target of opsonic antibodies (47) and that Tp0751 expression in model spirochetes results in its localization on the spirochete surface (43, 45).

T. pallidum is one of the few pathogens capable of crossing specialized endothelial barriers such as the retinal, placental, and blood-brain barriers (34–36, 38, 39). Other invasive pathogens that can cross the blood-brain barrier include Streptococcus pneumoniae, Neisseria meningitidis, and Haemophilus influenzae (50); these meningitis-causing organisms interact with a common cerebral endothelial cell receptor, the 67-kDa laminin receptor (LamR) (51), suggesting the existence of a common mechanism for attachment of extracellular bacterial pathogens to the brain microvasculature. LamR is a multifunctional protein that is converted from a 37-kDa precursor to the 67-kDa cell surface receptor via a mechanism that has yet to be elucidated (52). At the cell surface, LamR promotes tumor adhesion and migration (52–54), facilitates cell-ECM adhesion (55–59), and serves as a receptor for the prion protein (60), the Escherichia coli cytotoxic necrotizing factor (CNF1) (61, 62), select viruses (63–70), and neuroinvasive bacterial pathogens (51). In the current study, we explore T. pallidum dissemination mechanisms by investigating interactions between the Tp0751 adhesin and endothelial cells. We demonstrate that Tp0751 interacts with microvascular and macrovascular endothelial cells via the lipocalin fold-containing domain of the protein and that Tp0751-specific serum disrupts attachment of live T. pallidum to endothelial monolayers. We further identify LamR as an endothelial receptor for Tp0751 and present evidence suggesting that T. pallidum interacts with the same C-terminal region of LamR used by other neuroinvasive bacterial pathogens. This discovery suggests a possible common mechanism for vascular adhesion and extravasation of invasive, neurotropic extracellular bacterial pathogens.

## RESULTS

### Endothelial interactions are mediated by the C-terminal lipocalin fold-containing domain of Tp0751.

**FIG 1:**
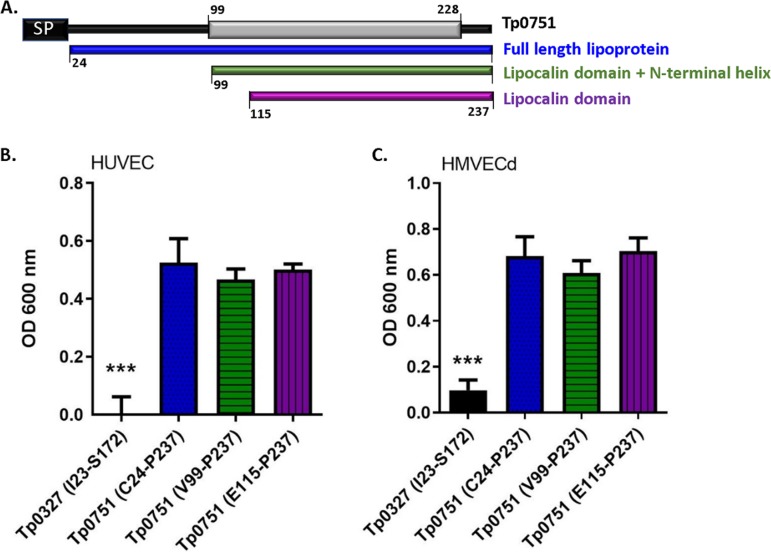
Endothelial binding is localized to the lipocalin fold-containing domain of Tp0751. (A) Schematic of Tp0751 primary sequence, including the signal peptide (SP) (black box) (M1 to S23 [M1–S23]), defined secondary structure elements (V99–H228) (gray box) (45), and N-terminal truncations corresponding to the mature lipoprotein (blue), the lipocalin fold-containing domain with the N-terminal helix (green), or the lipocalin fold-containing domain (purple). (B and C) Plate-based binding assays evaluated attachment of recombinant Tp0751 N-terminal truncations and the negative-control Tp0327 (I23–S172) to human umbilical vein endothelial cells (HUVECs) (B) and human microvascular dermal endothelial cells (HMVECd) (C). Proteins were added in equimolar concentration (25 μM), and results are presented as means plus standard errors of the means (SEM) (error bars) from three independent experiments performed in triplicate. Statistical analysis was performed by Student’s two-tailed *t* test comparing endothelial binding of Tp0751 N-terminal truncations to Tp0327 (I23–S172) (***, *P* < 0.001).

With the knowledge that Tp0751 can function as a vascular adhesin when expressed in a heterologous system (45, 48), we sought to further characterize endothelial interactions mediated by this T. pallidum protein. To delineate the endothelial binding interface of Tp0751, recombinant protein attachment to endothelial monolayers was evaluated using constructs with various N-terminal truncations. As shown in Fig. 1, endothelial binding was compared between Tp0751 (C24 to P237 [C24−P237]; mature lipoprotein sequence), Tp0751 (V99−P237; lipocalin fold plus N-terminal helix), and Tp0751 (E115−P237; lipocalin fold only). Plate-based binding assays were used to evaluate adhesion of Tp0751 truncations or a control treponemal protein, Tp0327, to confluent endothelial monolayers of microvascular and macrovascular origin. Recombinant proteins corresponding to all three N-terminal truncations of Tp0751 (C24−P237, V99−P237, and E115−P237) exhibited statistically significant binding to endothelial monolayers compared to the control recombinant protein, Tp0327 (Fig. 1B and C, *P* < 0.0001 by two-tailed Student’s *t* test). No difference in binding was observed between the three Tp0751 N-terminal truncation constructs (C24−P237, V99−P237, and E115−P237; Fig. 1B and C, not significant [ns] by two-tailed Student’s *t* test). These results demonstrate that endothelial binding localizes to the C-terminal lipocalin fold-containing domain of the protein.

### Tp0751 adheres to endothelial cells of microvascular and macrovascular origin.

**FIG 2:**
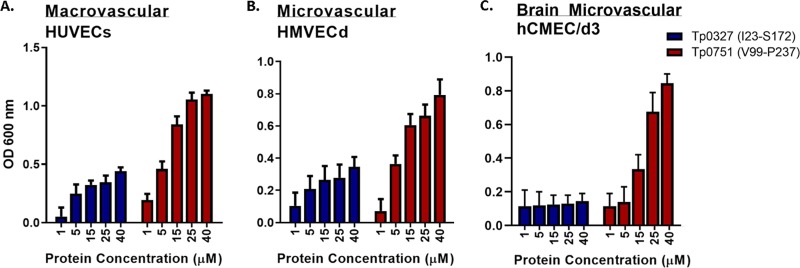
Tp0751 adheres to endothelial cells of microvascular and macrovascular origin. (A to C) Dose-dependent binding assays evaluating attachment of recombinant Tp0751 (V99–P237) or control recombinant Tp0327 (I23–S172) to host cell monolayers of macrovascular HUVECs (A), microvascular hMVECd (B), and human cerebral microvascular endothelial cells (hCMEC/d3) (C). Results are presented as mean optical density at 600 nm plus SEM (error bars) from triplicate wells in four independent experiments for HUVECs and hMVECd and from duplicate wells in two independent experiments for hCMEC/d3. Statistical analyses were performed by two-way ANOVA comparing endothelial binding of Tp0751 (V99–P237) to Tp0327 (I23–S172) in HUVECs (*P* = 0.017), hMVECd (*P* = 0.042), and hCMEC/d3 (*P* = 0.0033).

Attachment of Tp0751 to monolayers of endothelial cells of macrovascular and microvascular origin was evaluated using plate-based binding assays with a recombinant protein construct (Tp0751 [V99−P237]) that corresponds to the characterized structural domains of Tp0751 including the extended N-terminal helix and lipocalin fold-containing domain (45). Compared to the Tp0327 control protein, recombinant Tp0751 exhibited significant dose-dependent binding to two primary human endothelial cell lines: human umbilical vein macrovascular cells (HUVECs) (Fig. 2A, two-way analysis of variance [ANOVA], Tp0751 versus Tp0327, *P* = 0.017) and human microvascular dermal endothelial cells (HMVECds) (Fig. 2B, two-way ANOVA, Tp0751 versus Tp0327, *P* = 0.042). Further, Tp0751 displayed dose-dependent adhesion to a monolayer of immortalized cerebral brain microvascular endothelial cells (hCMEC/d3) (Fig. 2C, two-way ANOVA, Tp0751 versus Tp0327, *P* = 0.0033).

### Endothelial cell preexposure to Tp0751 enhances Tp0751-endothelial cell interactions.

**FIG 3:**
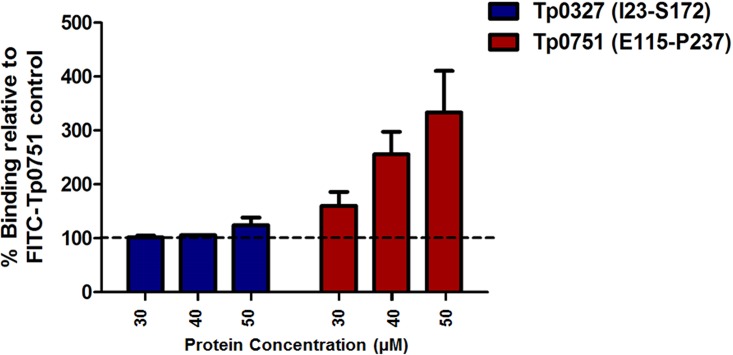
Preincubation of endothelial cells with Tp0751 enhances adhesin attachment. Binding assay evaluating attachment of FITC-labeled Tp0751 to endothelial cells following HUVEC preincubation with 30, 40, or 50 μM unlabeled Tp0751 (E115–P237) or control Tp0327 (I23–S172). Results are presented as means plus SEM from duplicate wells in two independent experiments and expressed as percent binding of the FITC-Tp0751-only control (no unlabeled protein added; set at 100%). Statistical analyses were performed by one-way ANOVA comparing binding of FITC-Tp0751 to endothelial monolayers preincubated with Tp0751 or negative-control Tp0327 (*P* = 0.004).

To further confirm the specificity of the Tp0751-endothelial cell interaction, we performed an additional competitive inhibition assay. In this experiment, HUVECs were preincubated with unlabeled Tp0751 or control Tp0327 prior to assessment of endothelial binding of FITC-labeled Tp0751. Intriguingly, preincubation of endothelial cells with unlabeled Tp0751 caused enhanced attachment of fluorescein isothiocyanate (FITC)-labeled Tp0751 in a dose-dependent manner (Fig. 3, two-way ANOVA, Tp0751 versus Tp0327, *P* = 0.004). Conversely, preincubation of endothelial cells with the unlabeled control protein, Tp0327, did not reduce the level of binding of FITC-labeled Tp0751 to endothelial cells, even when increasing amounts of Tp0327 were added to the endothelial cells. The results of this experiment confirm the specificity of the Tp0751-endothelial cell interaction and also suggest enhancement of endothelial binding upon prior incubation of endothelial cells with Tp0751.

### Tp0751-specific serum inhibits attachment of T. pallidum to endothelial cells.

**FIG 4:**
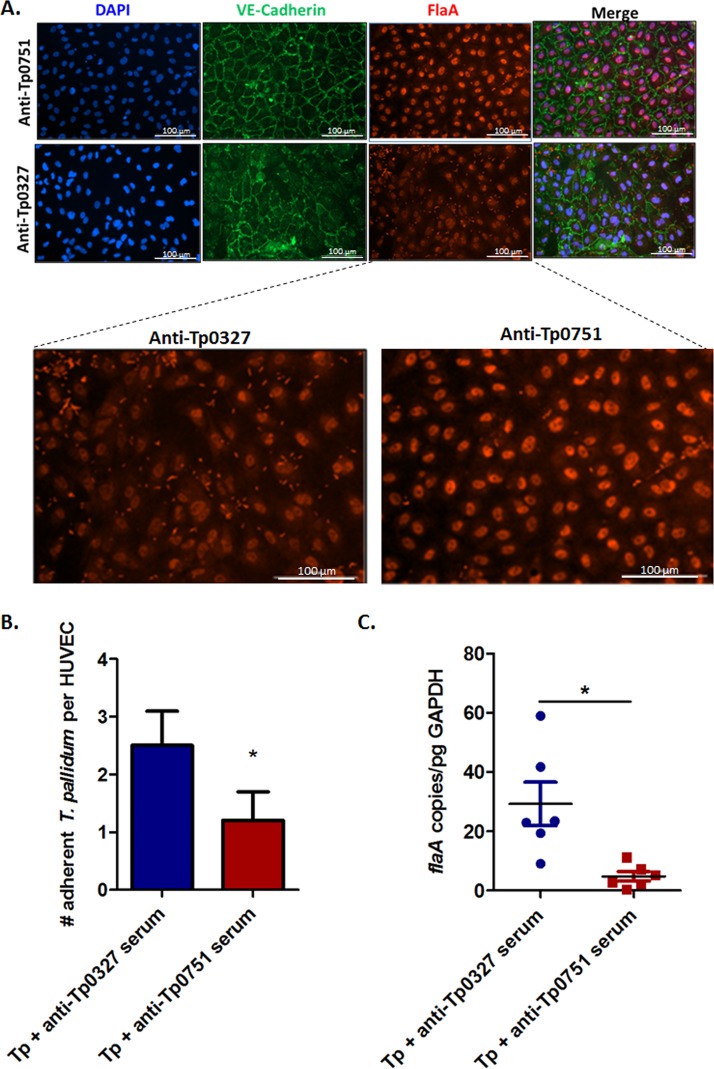
Tp0751-specific serum disrupts T. pallidum interactions with endothelial cells. (A) Representative immunofluorescent images from the T. pallidum serum inhibition adhesion assay showing HUVEC nuclei (blue; DAPI), cellular margins (green; anti-VE-cadherin), and T. pallidum (red; anti-FlaA). Quantification of HUVECs and T. pallidum were compared between anti-Tp0751 preincubations (top row) and anti-Tp0327 preincubations (bottom row). Zoomed images reveal comparisons of treponemal abundance (red; anti-FlaA) between anti-Tp0751 and anti-Tp0327 treatments. (B and C) Number of adherent T. pallidum (Tp) per HUVEC following preincubation with anti-Tp0327 serum (blue, negative control) or Tp0751-specific serum (red). In panel B, the mean number of T. pallidum per HUVEC plus SEM was quantified using immunofluorescence in a blind manner from five fields of view (FOV) in duplicate wells in four independent experiments. Statistical analysis was performed by Student’s two-tailed *t* test comparing endothelial binding of T. pallidum preincubated with Tp0751-specific serum to T. pallidum preincubated with Tp0327-specific serum (*P* = 0.025). In panel C, quantitative real-time PCR (qPCR) measured *flaA* DNA concentration from T. pallidum*-*endothelial adhesion assays. Results were normalized to HUVEC gDNA concentration by quantifying *GAPDH* and presented as mean *flaA* copy number per picogram of *GAPDH* DNA from duplicate wells in three independent experiments. Statistical analysis was performed by unpaired two-tailed *t* test with Welch’s correction comparing endothelial binding of T. pallidum preincubated with Tp0751-specific serum to T. pallidum preincubated with Tp0327-specific serum (*P* = 0.023).

To determine whether Tp0751 is involved in the endothelial attachment of live T. pallidum, we developed a T. pallidum adhesion assay where endothelial-treponemal interactions were detected and quantified via immunofluorescence imaging (see Fig. S1 and S2 in the supplemental material) or quantitative real-time PCR. Serum-mediated disruption of T. pallidum-endothelial interactions was evaluated by incubating T. pallidum with serum specific to Tp0751 or the control protein Tp0327, prior to coincubation with endothelial monolayers (Fig. 4A to C). Treponemes were maintained in an atmosphere of 34°C and 1.5 to 5% O_2_ to maximize treponemal health. Treponemal motility (an indicator of viability) was monitored throughout the experiment via dark-field microscopy to confirm the ongoing health of the organisms. Additionally, nonpermeabilized controls were incorporated into each experiment to validate T. pallidum outer membrane integrity by comparing reactivity of serum specific to the endoflagellar protein, FlaA, between permeabilized and nonpermeabilized samples (Fig. S3). In these experiments, treponemes preincubated with Tp0751-specific serum showed a statistically significant reduction in endothelial adhesion relative to T. pallidum incubated with the anti-Tp0327 serum control (Fig. 4B, Student’s two-tailed *t* test, *P* = 0.025; Fig. 4C, two-tailed *t* test with Welch’s correction, *P* = 0.023).

10.1128/mSphere.00195-20.1 FIG S1 Representative immunofluorescent images with cell quantification from the T. pallidum adhesion assay. HUVECs were quantified by automatic counting of nuclei (DAPI; blue; upper panel) via computational filtering within the size threshold of 10 to 60 μm (top right panel; yellow outline). Treponema pallidum (bottom left panel; inset) was quantified by automatic counting of treponemes (anti-FlaA; red) via computational filtering within the size threshold of 2 to 8 μm (bottom right panel; yellow outline). Microscopy images were captured with a Cytation 5 Imaging Reader, and automatic counting was conducted using the Cytation 5 software using size exclusion quantification settings. Download FIG S1, TIF file, 2.2 MB. Copyright © 2020 Lithgow et al. 2020 Lithgow et al. This content is distributed under the terms of the Creative Commons Attribution 4.0 International license.

10.1128/mSphere.00195-20.2 FIG S2 T. pallidum adhesion assay secondary antibody control. Representative epiimmunofluorescent image from the T. pallidum serum inhibition adhesion assay stained with secondary antibody only (no primary anti-FlaA antibodies) and DAPI (HUVEC nuclei), confirming specificity of the immunofluorescence staining. Microscopy images were captured with a Cytation 5 Imaging Reader. Download FIG S2, TIF file, 0.3 MB. Copyright © 2020 Lithgow et al. 2020 Lithgow et al. This content is distributed under the terms of the Creative Commons Attribution 4.0 International license.

10.1128/mSphere.00195-20.3 FIG S3 Validation of T. pallidum outer membrane integrity during endothelial attachment-inhibition assays. Samples taken from T. pallidum-endothelial attachment inhibition assays were fixed with 4% PFA in 8-well chamber slides and stained for the endoflagellar protein FlaA using anti-Tp0249 chicken serum and goat anti-chicken Alexa Fluor 568 to confirm that nonpermeabilized T. pallidum retained outer membrane integrity, as indicated by the absence of signal from FlaA immunofluorescence (panel A, column 2). These samples were compared to T. pallidum intentionally permeabilized with TX-100 to disrupt outer membrane integrity, allowing for visualization of treponemes by FlaA immunofluorescence (panel B, column 2). In each field of view, T. pallidum was also visualized by dark-field microscopy (panel A, columns 1 and 2). Representative epifluorescent and dark-field images obtained from a Nikon 80i fluorescence microscope at 1,000× magnification. Download FIG S3, TIF file, 0.4 MB. Copyright © 2020 Lithgow et al. 2020 Lithgow et al. This content is distributed under the terms of the Creative Commons Attribution 4.0 International license.

### Identification of LamR and stomatin as endothelial receptors for Tp0751.

To determine the molecular identity of host cell receptors targeted by Tp0751 during the process of vascular adhesion, we performed two independent affinity chromatography experiments using endothelial cells of macrovascular and microvascular origin, recombinant Tp0751, and mass spectrometry analysis (Fig. S4). This approach allowed an unbiased investigation of the Tp0751-targeted receptor(s) from the full repertoire of plasma membrane-associated proteins found in endothelial cells. Integral membrane proteins and membrane-associated proteins were isolated from HUVECs or hCMEC/d3 and incubated with recombinant Tp0751 immobilized on cobalt chelate columns. Interacting host proteins were eluted from the column and identified by liquid chromatography tandem mass spectrometry (LC-MS/MS). Data were refined to include only proteins identified in both independent experiments with greater than one significant peptide match from Tp0751-conjugated, but not control unconjugated, columns, and determined through literature analyses to localize to the surfaces of host cells. Based on these criteria, in two independent experiments using HUVECs and hCMEC/d3 as prey and Tp0751 (C24−P237) or Tp0751 (E115−P237) as bait, respectively, LamR and stomatin were identified as candidate receptors for Tp0751 (Table 1; see also Tables S1 and S2 in the supplemental material).

**TABLE 1:** Candidate endothelial receptors for Tp0751 identified by affinity chromatography paired with tandem mass spectrometry

Endothelial membrane protein	Cell^*a*^	No. of peptides^*b*^	Peptide sequence
67-kDa laminin receptor	HUVECs	4	(K)SDGIYIINLK(R)
			(R)FTPGTFTNQIQAAFR(E)
			(R)AIVAIENPADVSVISSR(N)
			(R)ADHQPLTEASYVNLPTIALCNTDSPLR(Y)

	hCMEC/d3	4	(K)FLAAGTHLGGTNLDFQMEQYIYK(R)
			(R)AIVAIENPADVSVISSR(N)
			(K)FAAATGATPIAGR(F)
			(R)FTPGTFTNQIQAAFR(E)

Stomatin^*c*^	HUVECs	5	(R)ILQGGAKGPGLFFILPCTDSFIKVDMR(T)
			(R)ALKEASMVITESPAALQLR(Y)
			(K)EASMVITESPAALQLR(Y)
			(K)NSTIVFPLPIDMLQGIIGAK(H)
			(R)VQNATLAVANITNADSATR(L)

	hCMEC/d3	2	(K)EASMVITESPAALQLR(Y)
			(R)VQNATLAVANITNADSATR(L)

a HUVECs are macrovascular cells, and hCMEC/d3 are brain microvascular cells.

b Peptides found exclusively in the bait-prey sample; >1 unique, significant peptide hit; localization to the external face of the plasma membrane of host cells.

c Erythrocyte band 7 integral membrane protein.

10.1128/mSphere.00195-20.4 FIG S4 Schematic illustration of the affinity chromatography mass spectrometry approach for identification of candidate endothelial cell receptors for Tp0751. Recombinant histidine-tagged Tp0751 was immobilized to a cobalt chelate matrix, followed by the addition of membrane proteins and membrane-associated proteins isolated from endothelial cells. Following a series of incubations and washes, interacting proteins were eluted with imidazole and identified by liquid chromatography tandem mass spectrometry from two separate experiments (differences in the protocol denoted in blue or green). In parallel, an unconjugated cobalt chelate affinity column was subject to the same conditions to identify endothelial proteins that interact nonspecifically with the chelate. Candidate endothelial receptors were identified from peptides that were found exclusively in the bait-prey sample in both experiments, had greater than one unique significant peptide hit, and were known to localize to the surface-exposed host membrane. Download FIG S4, TIF file, 0.3 MB. Copyright © 2020 Lithgow et al. 2020 Lithgow et al. This content is distributed under the terms of the Creative Commons Attribution 4.0 International license.

10.1128/mSphere.00195-20.6 TABLE S1 Tp0751 (E115-P237)-interacting hcMEC/d3 integral membrane and membrane-associated proteins identified by affinity chromatography and mass spectrometry. Download Table S1, DOCX file, 0.01 MB. Copyright © 2020 Lithgow et al. 2020 Lithgow et al. This content is distributed under the terms of the Creative Commons Attribution 4.0 International license.

10.1128/mSphere.00195-20.7 TABLE S2 Tp0751 (C24-P237)-reactive HUVEC integral membrane and membrane-associated proteins identified by affinity chromatography and mass spectrometry. Download Table S2, DOCX file, 0.01 MB. Copyright © 2020 Lithgow et al. 2020 Lithgow et al. This content is distributed under the terms of the Creative Commons Attribution 4.0 International license.

### Recombinant Tp0751 interacts with endogenous LamR from cerebral brain endothelial cells.

**FIG 5:**
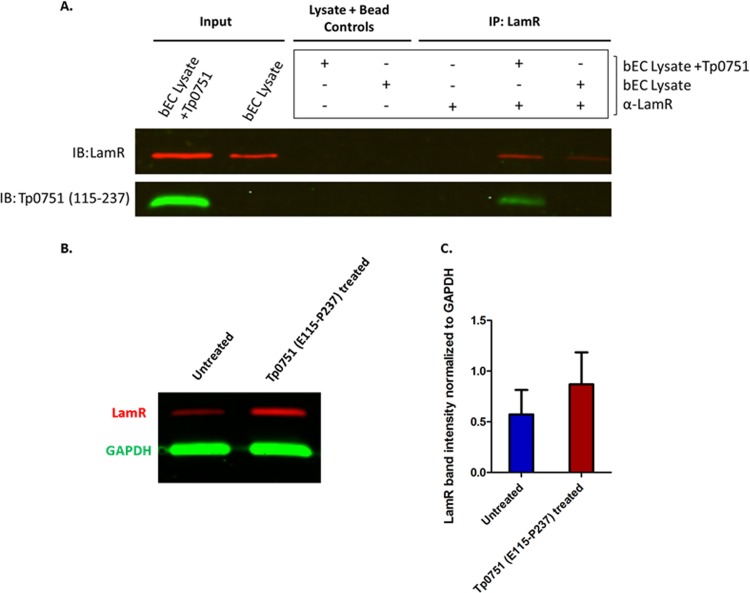
Recombinant Tp0751 interacts with endogenously expressed LamR in brain endothelial cells (bECs). Live hCMEC/d3 monolayers were left untreated (bEC lysate) or coincubated with exogenous recombinant Tp0751 (bEC Lysate + Tp0751). (A) Lysates were incubated with magnetic beads in the absence of antibodies (Lysate + Bead Controls) to control for nonspecific interactions between LamR and the beads. LamR was immunoprecipitated (IP) from lysates using mouse anti-LamR antibody (α-LamR) covalently coupled to magnetic beads, and coimmunoprecipitation of Tp0751 was evaluated by immunoblotting (IB) against Tp0751 (aa 115–237) and LamR. Representative results from three independent experiments are shown. (B) Quantitative immunoblot of LamR (red) and internal control protein GAPDH (green) from untreated or Tp0751-treated bEC lysates (C) LamR band intensity normalized to GAPDH band intensity from untreated or Tp0751-treated bEC lysates. Results representative of two independent experiments.

The molecular interaction between Tp0751 and LamR was validated using coimmunoprecipitation. Following incubation of recombinant Tp0751 (E115−P237) with live hCMEC/d3 monolayers, specific binding between Tp0751 and endogenous LamR was confirmed by demonstrating that Tp0751 coprecipitates with LamR when LamR-specific antibodies are covalently linked to magnetic beads (Fig. 5A). An enrichment of LamR of at least 12-fold was observed in coimmunoprecipitations (Fig. 5A, IP: LamR) compared to controls where lysates were incubated with magnetic beads in the absence of antibodies (Fig. 5A, Lysate + Bead Controls). Notably, the immunoblot reveals that LamR is more abundant in lysates from samples treated with Tp0751 compared to the untreated control (Fig. 5A, Input, IP), suggesting that Tp0751 may cause upregulation of LamR. In support of this finding, a quantitative immunoblot of LamR normalized to the internal control protein glyceraldehyde 3-phosphate dehydrogenase (GAPDH) revealed that hCMEC/d3 cells treated with 25 μM Tp0751 for 60 min have approximately 1.5-fold-higher LamR protein levels than untreated cells (Fig. 5B and C). These findings provide support for the results of the competitive inhibition assay reported in Fig. 3. In this assay, preincubation of endothelial cells with a range of 30 to 50 μM Tp0751 for 90 min led to increasing attachment of FITC-labeled Tp0751 in a manner that paralleled the Tp0751 preincubation dose. This suggests that exposure of endothelial cells to increasing amounts of Tp0751 caused a corresponding increase in expression of LamR, which in turn bound more FITC-labeled Tp0751.

### Confirmation of the specificity of the Tp0751-LamR interaction using plate-based binding assays.

**FIG 6:**
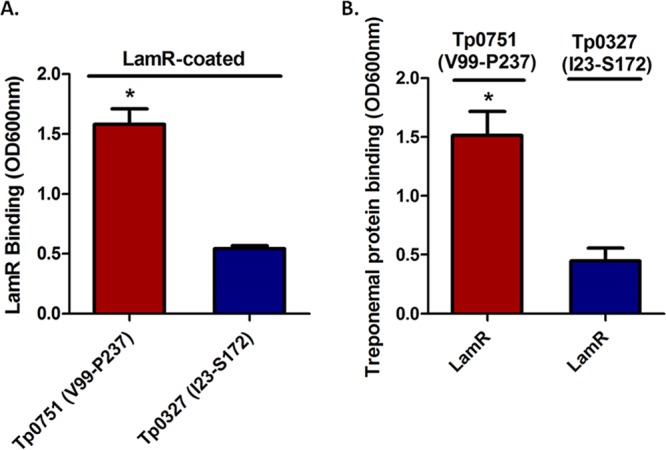
Reciprocal binding between recombinant Tp0751 and LamR. (A) Binding of recombinant Tp0751 (V99–P237) or control treponemal protein Tp0327 (I23–S172) to immobilized LamR. (B) Binding of recombinant LamR to immobilized Tp0751 (V99–P237) or Tp0327 (I23–S172). Results are presented as mean optical density at 600 nm plus SEM from duplicate wells in three independent experiments for LamR-immobilized binding assays and from duplicate wells in two independent experiments for Tp0751-immobilized binding assays. Statistical analyses were performed by Student’s two-tailed *t* test comparing binding of Tp0751 and Tp0327 to immobilized LamR (*P* = 0.0157) and LamR binding to immobilized Tp0751 versus immobilized Tp0327 (*P* = 0.0447).

To further confirm the existence of a specific interaction between Tp0751 and LamR, we performed a reciprocal plate-based binding assay using recombinant versions of Tp0751 and LamR. As shown in Fig. 6A, Tp0751 exhibited statistically significant higher levels of binding to LamR-coated wells compared to the binding of negative-control treponemal protein Tp0327 (*P* = 0.0157). Further to this, in a reciprocal binding assay, recombinant LamR displayed significantly higher binding to immobilized Tp0751 compared to immobilized Tp0327 (Fig. 6B, *P* = 0.0447).

### T. pallidum interacts with endogenous LamR in cerebral brain endothelial cells.

**FIG 7:**
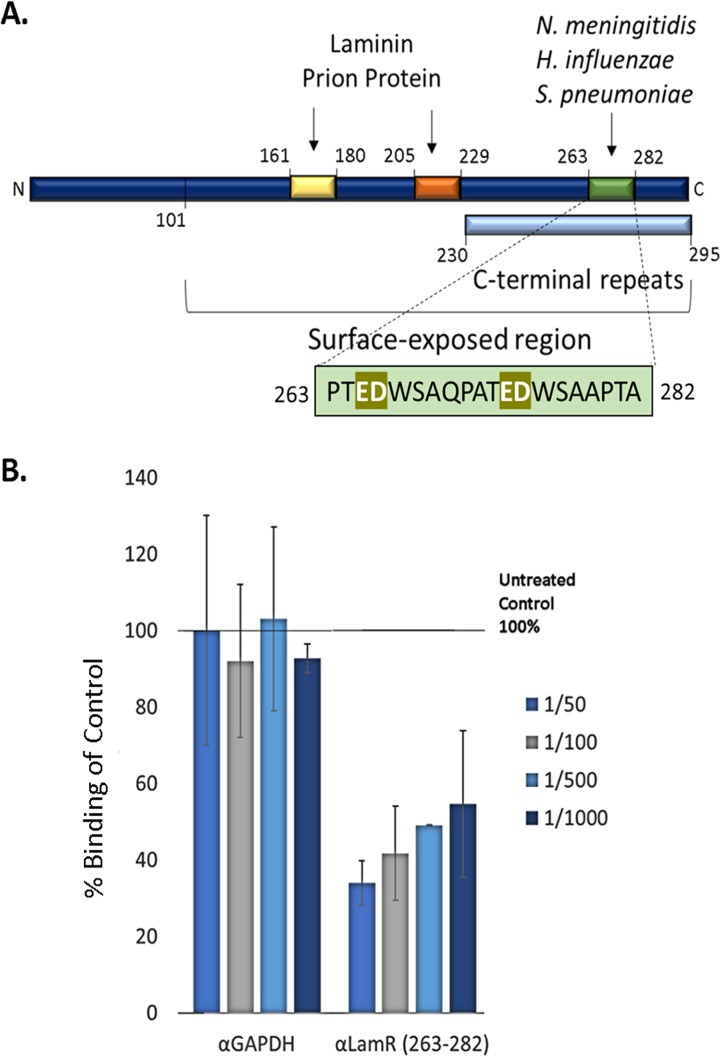
T. pallidum interacts with LamR (aa 263–282) on brain endothelial cell surfaces. (A) Schematic illustration of characterized interaction regions of LamR (51, 71, 72, 74, 75). Elements include the predicted surface-exposed region (aa 101–295), C-terminal repeats (light blue box, aa 230–295), binding sites for laminin and the prion protein, including the peptide G region (yellow box; aa 161–180) and direct binding region (orange box; aa 205–229) and the neuroinvasive bacterial pathogen binding site (green box; aa 263–282). (B) T. pallidum attachment to brain endothelial cells (hCMEC/d3) was evaluated using immunofluorescence detection of adherent treponemes (FlaA; red) and hCMEC/d3 nuclei (DAPI; blue) at ×200 magnification from five FOV in duplicate from two independent experiments. Inhibition of T. pallidum attachment to hCMEC/d3 cells by antibodies was quantified as a percentage of the untreated no-antibody control (set at 100%). Results are presented as means ± SEM, and statistical analysis was performed by two-way ANOVA comparing T. pallidum binding to endothelial cells following hCMEC/d3 preincubation with anti-GAPDH (α-GAPDH) versus anti-LamR (aa 263–282) where *P* = 0.0037.

LamR contains three distinct interaction regions that are engaged by laminin (71–74), prion protein (72, 75), and the neuroinvasive pathogens S. pneumoniae, H. influenzae, and N. meningitidis (51) (Fig. 7A). The peptide G region (Fig. 7A, yellow box, amino acids 161 to 180 [aa 161–180]) and direct binding site (Fig. 7A, orange box, aa 205–229) are bound by laminin and the prion protein (71–75), whereas a discrete region of the LamR C terminus (Fig. 7A, green box, aa 263–282) is the attachment site for several meningitis-causing bacterial pathogens (51). Given the neuroinvasive capability of T. pallidum, we utilized our T. pallidum*-*endothelial adhesion assay (Fig. 4) to determine whether attachment to brain endothelial cells could be disrupted by blocking the LamR C-terminal pathogen binding region. Polyclonal antibodies specific to the LamR C terminus (aa 263–282) inhibited T. pallidum attachment to hCMEC/d3 cells in a dose-dependent manner (Fig. 7B, *P* = 0.0037, two-way ANOVA), while a negative-control polyclonal antibody specific to the irrelevant protein GAPDH had no effect on T. pallidum endothelial attachment at all antibody dilutions tested (Fig. 7B). Immunofluorescence experiments conducted under the same culture conditions provided support for surface expression of LamR in endothelial cells (Fig. S5). Although this experimental result presents supporting evidence for the interaction of T. pallidum with the neuroinvasive pathogen binding site of LamR, we cannot at this point rule out the possibility that steric hindrance originating from the antibodies reduced attachment of T. pallidum to endothelial cells in a LamR-dependent, but pathogen binding site-independent, manner.

10.1128/mSphere.00195-20.5 FIG S5 Localization of LamR in cultured endothelial cells. Endothelial monolayers of HUVECs (A) and hCMEC/d3 (B) grown under the same experimental culture conditions were fixed with 4% PFA and stained with a LamR antibody (red) and a nuclear stain (DAPI, blue). Visualization of LamR from nonpermeabilized monolayers suggests surface localization of LamR. Validation that cell membranes remained intact was achieved using GAPDH antibody (green) to detect the protein in nonpermeabilized (C) and permeabilized (D) endothelial monolayers. Representative epifluorescent images obtained from a Cytation 5 imager at 200× magnification. Download FIG S5, TIF file, 2.7 MB. Copyright © 2020 Lithgow et al. 2020 Lithgow et al. This content is distributed under the terms of the Creative Commons Attribution 4.0 International license.

### The previously identified endothelial binding region of Tp0751, encompassing aa 172–195, interacts with LamR.

**FIG 8:**
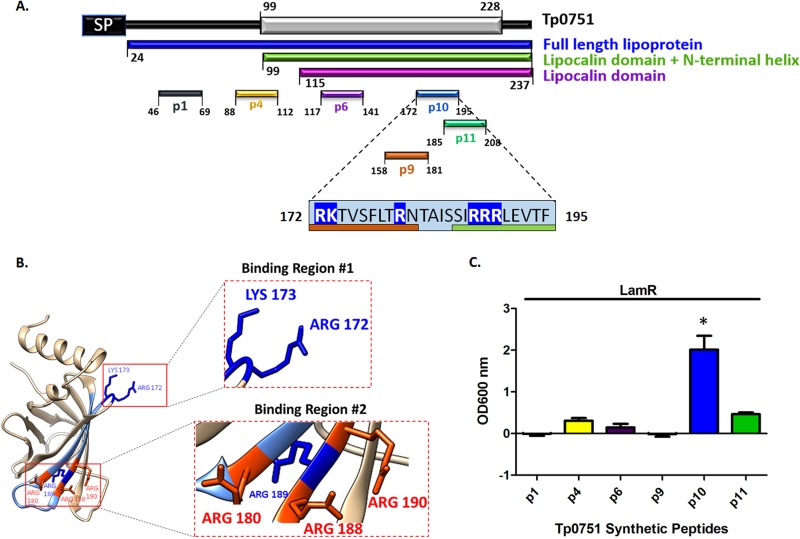
LamR interaction region of Tp0751. (A) An N-terminal Tp0751 peptide (p1 [aa 46–69], peptides from ECM-binding regions of Tp0751 (p4 [aa 88–112], p6 [aa 117–141]), a peptide corresponding to the putative Tp0751 endothelial interaction region (p10 [aa 172–195]) (45), and peptides overlapping the endothelial interaction region (p9 [aa 158–181], p11 [aa 185–208]) are shown within the primary amino acid sequence. In the light blue box showing aa 172–195, positively charged residues are shown on royal blue background, residues contained within neighboring peptides are indicated by an orange bar below the residues (p9 overlapping residues) or by a green bar below the residues (p11 overlapping residues). (B) Tp0751 structural model (interaction region, light blue) revealing two positively charged regions shown as insets of the ribbon diagram. Binding Region #1 is a looped structure with positively charged amino acids displayed in blue (R172 and K173). Binding Region #2 is a clustered region of exposed positively charged amino acids displayed in red (R180, R188, and R190). (C) Binding of Tp0751 synthetic peptides to immobilized LamR. Tp0751 synthetic peptides corresponding to the non-ECM-binding control peptide p1 (aa 46–69), ECM-binding peptides p4 (aa 88–112), p6 (aa 117–141), p10 (aa 172–195), p11 (aa 185–208), and p9 (aa 158–181) that share positively charged residues with p10. Results are presented as mean optical density at 600 nm plus SEM from duplicate wells in two independent experiments, and statistical analysis was performed by two-way ANOVA with Tukey’s *post hoc* analysis comparing p10 binding with binding of all other Tp0751 synthetic peptides (*P* < 0.05).

Peptide inhibition studies previously performed with a Tp0751-expressing B. burgdorferi heterologous expression system suggest that the endothelial binding capacity of Tp0751 is localized to a discrete region corresponding to aa 172–195 within the lipocalin fold-containing domain of Tp0751 (45), a finding consistent with our observations from the current study (Fig. 1). The primary sequence of the proposed Tp0751 endothelial interaction region features six positively charged residues (Fig. 8A, R172, K173, R180, R188, R189, and R190). Mapping of the endothelial interaction region (45) onto the Tp0751 structure localized two positively charged residues to an exposed loop region of the beta barrel (Fig. 8B, blue, R172 and K173) and three exposed positively charged residues to a region in the antiparallel beta sheets (Fig. 8B, red, R180, R188, and R190). Prior attempts at producing recombinant versions of Tp0751 with site-directed mutagenesis of key residues in the proposed endothelial binding region encompassing residues 172–195 have been unsuccessful, likely because the mutations disrupted the tight beta barrel of Tp0751. To determine whether this region of Tp0751 also interacts with LamR, we instead performed a plate-based binding assay using synthetic Tp0751 peptides (42) and immobilized recombinant LamR. A statistically significant level of binding was observed between the Tp0751 p10 peptide covering aa 172–195 and LamR versus all other Tp0751 peptides tested (Fig. 8C, one-way ANOVA with Tukey’s *post hoc* comparison, *P* < 0.05). Conversely, no significant LamR binding was observed with a control Tp0751 peptide (p1 encompassing aa 46–69), peptides previously shown to bind to extracellular matrix components (p4 [aa 88–112], p6 [aa 117–141], and p11 [aa 185–208]) (42), or peptides partially overlapping the proposed endothelial binding region (p9 [aa 158–181] and p11 [aa 185–208]) (45) in the plate-based binding assay. This experiment provides supporting evidence that the interaction of Tp0751 aa 172–195 with endothelial cells (45) arises from interaction of this region with LamR. This experiment further suggests that a combination of the proposed binding regions 1 and 2 (Fig. 8B) are required for LamR attachment, as binding region 1 or 2 alone (in p9 and p11, respectively) do not confer significant binding to LamR (Fig. 8C).

## DISCUSSION

The highly invasive nature of T. pallidum is illustrated by its widespread vascular dissemination during early disease progression, with spirochetes traversing across tissue barriers as well as highly protected blood-brain, placental, and retinal barriers (23, 24, 34, 35, 38, 39). Treponema pallidum*-*endothelial interactions are a critical step in this process, but elucidation of the molecular mechanisms for vascular adhesion has been limited by the challenges associated with studying T. pallidum. Our investigations provide evidence confirming that Tp0751 is a vascular adhesin that interacts with microvascular and macrovascular endothelial cells, including cerebral brain endothelial cells. We also identify an endothelial receptor for Tp0751 that is targeted by other extracellular neuroinvasive bacterial pathogens, suggesting the existence of a shared invasive strategy among neurotropic pathogens.

It has been well established that T. pallidum binds to host-derived ECM components (76) as well as a variety of mammalian cell types (77–79), including endothelial cells (80–82), and numerous ECM-binding interactions with specific T. pallidum proteins have been confirmed using *in vitro* assays with live T. pallidum (42, 83, 84). Herein we report the direct demonstration of disruption of a T. pallidum-host endothelial interaction using Tp0751-specific antiserum. We also demonstrate that Tp0751 adheres to cell lines of diverse origin, suggesting that Tp0751 may mediate vascular adhesion in both the macrovasculature and microvasculature of the host. Further, we show attachment of Tp0751 to the immortalized blood-brain barrier model cell line hCMEC/d3, an important first step in investigating the contribution of this adhesin to the process of T. pallidum neuroinvasion. Taken in the context of our previous findings, which demonstrate that Tp0751 functions as a vascular adhesin *in vitro* and *in vivo* when expressed heterologously in a model spirochete (45, 48), these results present evidence using three distinct approaches that Tp0751 is an endothelial adhesin.

Structural characterization of Tp0751 has revealed that the C-terminal domain adopts a lipocalin fold with a unique extended N-terminal alpha helix and that attachment to ECM components is mediated by four host binding regions that map along a single face of the protein, including these two structural regions (45). It is predicted that these four binding regions synergistically coordinate interactions with large ECM components (42, 45). A competitive inhibition assay using the B. burgdorferi heterologous expression system and synthetic peptides from known Tp0751 ECM-binding regions spanning aa G88−A112, Q117−I141, R172−F195, and S185−V208 demonstrated that the endothelial binding capacity of Tp0751 is localized to a discrete region corresponding to aa R172−F195 (45). In this study, we demonstrate that the endothelial binding capacity of Tp0751 localizes to the C-terminal lipocalin fold-containing domain, since identical levels of endothelial binding were observed for three N-terminal truncations of Tp0751 that contained the lipocalin fold. This supports our previous finding that the endothelial binding of Tp0751-expressing B. burgdorferi is localized to a defined region within the lipocalin fold (45). Collectively, these results suggest the possibility that Tp0751 interacts with a specific endothelial cell receptor via a discrete region of the lipocalin fold, rather than mediating contact with the vasculature through an ECM bridge, since the N-terminal helix is not required for recombinant Tp0751-host cell interactions and only one out of four of the ECM-binding Tp0751 peptides competitively inhibited Tp0751-mediated spirochete attachment to endothelial cells (45).

To further characterize Tp0751 interactions with vascular surfaces, we utilized an unbiased affinity chromatography approach coupled with mass spectrometry, with the primary aim of identifying candidate endothelial receptors targeted by Tp0751. This approach identified LamR and stomatin (85, 86) as interaction partners for Tp0751 from two independent experiments using two different endothelial cell lines; as a fuller body of literature exists for the role of LamR in endothelial cells, we focused our attention on this host cell receptor. LamR exists in two distinct forms within host cells: (i) a low-molecular-weight 37-kDa laminin receptor precursor (LRP) found primarily in intracellular locations and (ii) a high-molecular-weight 67-kDa laminin receptor (LamR) that localizes to cell surfaces (52). The mechanisms underlying LRP conversion to the high-molecular-weight 67-kDa LamR species and localization to the cell surface remain unknown. However, LamR is thought to localize to lipid rafts at the cell surface (87, 88), and the current hypotheses for precursor conversion to the high-molecular-weight form of LamR include (i) a large posttranslational modification (such as SUMOylation), (ii) homodimerization, or (iii) heterodimerization (52).

In light of these unanswered questions regarding LamR composition and the mechanism of cell surface localization, we used two independent experimental strategies to confirm the Tp0751-LamR interaction, incorporating both endogenous and exogenous sources of LamR. The first confirmatory experiment validated that recombinant versions of LamR and Tp0751 interacted in reciprocal plate-based binding assays. The second confirmatory experiment explored the interactions of Tp0751 with endogenous LamR in the relevant context of endothelial cells, rather than using a LamR depletion approach which has been previously shown to negatively affect cell viability (89–93). Since the initial identification of receptors for Tp0751 relied upon immobilization of the adhesin to isolate interacting endothelial proteins, we used the opposite format in coimmunoprecipitation to confirm binding between Tp0751 and LamR. In this approach, endogenous LamR was immobilized from lysates of brain endothelial cell monolayers that had been incubated with exogenous recombinant Tp0751. This antibody-based purification of endogenous LamR resulted in coprecipitation of Tp0751 from the mixture. Intriguingly, in three independent experiments, a higher abundance of LamR was observed in both the “Input” and “IP” lanes of the immunoblot from Tp0751-treated lysates compared to control lysates. This result is further supported by the observation that preincubation of endothelial cells with unlabeled Tp0751 enhanced attachment of FITC-labeled Tp0751 in a dose-dependent manner. Specifically, exposure of endothelial cells to 25 μM Tp0751 led to an approximately 1.5-fold increase in LamR expression (Fig. 5), consistent with the observation that preexposure of endothelial cells to 30 μM unlabeled Tp0751 led to approximately 150% increased attachment of FITC-labeled Tp0751 to endothelial cells (compared to endothelial cells that had not been preexposed to unlabeled Tp0751; Fig. 3). These results suggest that Tp0751 exposure to endothelial cells induces upregulation of LamR surface expression.

Collectively, these investigations have verified the LamR-Tp0751 interaction using five independent experimental techniques. These techniques include the following: (i) affinity chromatography with recombinant Tp0751 and endogenous LamR from human brain endothelial cells, with in-solution digestion and mass spectrometry analysis; (ii) affinity chromatography with recombinant Tp0751 and endogenous LamR from human macrovascular endothelial cells, with in-gel digestion and mass spectrometry analysis; (iii) coimmunoprecipitation using recombinant Tp0751 and endogenous LamR from cerebral brain microvascular endothelial cells; (iv) binding assays using recombinant Tp0751 and immobilized recombinant LamR; and (v) binding assays using recombinant LamR and immobilized recombinant Tp0751. To extend these results to T. pallidum, and specifically to validate the interaction between T. pallidum and LamR in the biologically relevant context of an endothelial cell membrane, we utilized an antibody blocking approach with polyclonal antibodies specific to the LamR pathogen binding site (51). LamR contains a discrete site within the C-terminal region (aa 263–282) that is targeted by three meningitis-causing bacteria (51). The C-terminal region of LamR is inherently disordered (94) and contains an abundance of acidic residues and five (D/E)W(S/T) repeats (52, 72), two of which are found within the LamR pathogen binding site (51). Antibodies directed to the LamR pathogen binding site disrupted T. pallidum interactions with cerebral microvascular endothelial cells, suggesting that T. pallidum, similar to other neuroinvasive pathogens, may target the pathogen binding site of LamR. Of relevance to the current study, the previously characterized putative endothelial interaction region of Tp0751 (45) is highly positively charged, with hydrophobic residues localized within two exposed basic patches in the lipocalin fold (Fig. 8A and B). The bacterial ligands responsible for LamR engagement in N. meningitidis, H. influenzae, and S. pneumoniae were identified as PilQ and PorA, OmpP2, and CbpA, respectively (51). The LamR-binding motifs for CbpA (EPRNEEK) and OmpP2 (RNSKNDAGWG) both map to exposed loop regions of the adhesin structures (51, 95) but do not share an obvious consensus sequence with each other or the Tp0751 host binding region (Fig. 8). The negatively charged nature of the LamR pathogen binding site (Fig. 7A) and the abundance of positively charged residues localized to exposed regions within the Tp0751 endothelial binding site (Fig. 8) suggest that electrostatic interactions could underlie the LamR-Tp0751 molecular interface. The similarity of endothelial binding between Tp0751 N-terminal truncations, which have different net charges, suggests that the overall charge of the adhesin does not drive receptor interactions. In the current study, we used a plate-based binding assay to demonstrate that a specific charged region of Tp0751 corresponding to amino acids 172–195 bound to LamR, providing evidence in support of the concept of a discrete region of Tp0751 interacting directly with LamR.

Prior investigations have demonstrated that T. pallidum and Tp0751 have the capacity to interact with laminin (42, 46, 48, 76); therefore, it is possible that a laminin bridge may also contribute to the Tp0751-LamR interaction. The binding assays using recombinant Tp0751, synthetic Tp0751 peptides, and recombinant LamR provide evidence for a direct interaction between Tp0751 and LamR. Additional evidence from the current study also indicates a direct LamR-Tp0751 interaction. If laminin were required for this interaction, we would anticipate coisolating laminin peptides with LamR during affinity chromatography, yet no laminin peptides were detected during mass spectrometry analyses in either of the two experiments. Further to this, T. pallidum attachment to endothelial cells was disrupted with antibodies specific to the LamR C-terminal pathogen binding region (aa 263 − 282), suggesting that this region of LamR is mediating the interaction. While findings from LamR peptide-based approaches have led researchers to hypothesize that the negatively charged C-terminal repeats in LamR are involved in laminin binding (72), evidence exists that counters this hypothesis, including the following: (i) a LamR mutational study that revealed that F32, E35, and R155 are the residues required for laminin binding (96) and (ii) a gain-of-function study that demonstrated the introduction of a LamR C-terminal construct into the non-laminin-binding Archaeoglobus fulgidus failed to confer laminin binding capacity (52). Thus, to date, there is no convincing evidence to show that the LamR pathogen binding site mediates binding to laminin, and our investigations suggest a direct interaction between T. pallidum and the LamR C terminus. Future investigations will need to further probe whether laminin, via an interaction with the laminin-binding region of LamR, could play a role in these complex interactions.

A growing body of evidence has revealed diverse roles for Tp0751 in the vasculature from endothelial cell adhesion (45, 48) to basement membrane engagement (42, 43). With the knowledge that Tp0751 can interact with LamR on endothelial surfaces as well as ECM components found within the vasculature, including fibronectin and fibrinogen (42–44, 46, 47), it appears that Tp0751 participates in a variety of molecular interactions during dissemination. On the basis of our current findings, we hypothesize that initial T. pallidum interactions with vascular surfaces are mediated by tethering interactions between Tp0751 and ECM components such as fibronectin. Subsequent interactions would involve stationary adhesion in which Tp0751 interacts directly with LamR, similar to the process revealed for other neuroinvasive pathogens (51). A more robust understanding of T. pallidum interactions with endothelial cells will be essential for elucidating the underlying mechanisms for dissemination of this pathogen. Such investigations will provide mechanistic insight into pathogenic strategies shared by highly invasive pathogens that supersedes traditional bacterial taxonomic classification.

## MATERIALS AND METHODS

### Ethics statements.

All animal studies were approved by the local institutional review board at the University of Victoria under protocol 2016-033 and were conducted in strict accordance with standard accepted principles as set forth by the Canadian Council on Animal Care (CCAC), National Institutes of Health, and the U.S. Department of Agriculture in facilities accredited by the American Association for the Accreditation of Laboratory Animal Care and the CCAC.

### Cloning and purification of recombinant proteins.

The genes encoding N-terminal His_6_ forms of the treponemal proteins Tp0327 (I23−S172), the full-length mature form of Tp0751 (Tp0751 [C24−P237]), and Tp0751 N-terminal truncations (Tp0751 [V99−P237] and Tp0751 [E115−P237]) were cloned as previously described (45, 46, 97). Tp0327 and Tp0751 constructs were purified from Escherichia coli BL21-AI or BL21(DE3), subjected to nickel affinity chromatography with HisTrap FF columns (GE Healthcare, Mississauga, Ontario, Canada), and further purified with size exclusion/cation exchange chromatography (HiLoad 16/60 Superdex 75; GE Healthcare) on an AKTA Prime Plus FPLC system (GE Healthcare) in a final buffer of 20 mM HEPES (pH 7.0), 150 mM NaCl, and 1% glycerol as previously described (45, 46, 97).

### Recombinant protein FITC labeling.

Recombinant Tp0751 (V99−P237) was purified as described above except all buffers were prepared without glycerol. Following purification, Tp0751 (V99−P237) was chemically labeled with fluorescein isothiocyanate isomer I (FITC I; SigmaAldrich, Oakville, Ontario, Canada). Five milligrams of recombinant protein were incubated with 0.5 mg of FITC in a final volume of 5 ml of 1 M sodium bicarbonate (pH 9.1) for 1 h at room temperature (RT). Samples were then buffer exchanged into 20 mM HEPES, 150 mM NaCl (pH 7.0) using a 10,000-kDa molecular-weight cutoff (MWCO) Centricon (Millipore, Etobicoke, Ontario, Canada) to remove unconjugated FITC.

### Endothelial cell culturing.

Human umbilical vein endothelial cells (HUVECs) (Lonza, Mississauga, Ontario, Canada) were cultured as previously described (45). Human dermal microvascular endothelial cells (HMVECds) from pooled donors (Lonza) and human brain endothelial cells hCMEC/d3 (Millipore) were cultured at 37°C in an atmosphere of 5% CO_2_ in endothelial growth medium-2 microvascular (EGM-2 MV) and EndoGro (Millipore, Etobicoke, Ontario, Canada) supplemented with basic fibroblast growth factor (Millipore), respectively.

### Treponema pallidum propagation.

Treponema pallidum subsp. *pallidum* (Nichols strain) was propagated in, and extracted from, New Zealand White rabbits as previously described (98). Testicular extractions were performed with three consecutive extractions for 10, 30, and 60 min in 10% normal rabbit serum (NRS) in 0.9% NaCl at RT in a modified anaerobic chamber with an atmosphere of 1.5 to 5% O_2_ and 5% CO_2_ balanced with N_2_ (Coy Laboratories, Grass Lake, MI). Oxygen levels were monitored using a LabQuest2 oxygen meter (Vernier, Beaverton, Ontario, Canada). Gross cellular debris was removed by centrifuging the extractions at 220 × *g* at RT for 5 min.

### Endothelial cell attachment assays using recombinant treponemal proteins.

Equal seeding densities of endothelial cells (1.5 × 10^4^ cells/well) were added to 96-well plates (Sarstedt, Sarstedt, Germany). Cells were grown for 48 h at 37°C in 5% CO_2_ to form confluent monolayers. Endothelial cells were washed with warm HEPES-buffered saline (HBSS) (Lonza, Mississauga, Ontario, Canada) and incubated with recombinant negative-control protein Tp0327 or recombinant Tp0751 proteins for 90 min at 37°C in an atmosphere of 5% CO_2_. To calculate the concentration of the recombinant proteins, UV absorbance at 280 nm was measured prior to each experiment using a spectrophotometer. Molar concentrations of proteins were calculated based on the molecular weight of each recombinant protein in a defined volume to ensure the addition of equimolar amounts of protein. Monolayers were gently washed three times with warm HBSS to remove nonadherent protein, fixed in 2% paraformaldehyde (PFA), and blocked for 30 min in 1% bovine serum albumin (BSA) in phosphate-buffered saline (PBS) at 37°C in an atmosphere of 5% CO_2_. Attachment of recombinant proteins to monolayers was evaluated from triplicate wells that had been seeded with equal numbers of endothelial cells by detecting the relative signal from N-terminal hexahistidine tags on recombinant proteins compared to no-protein controls using nickel-labeled horseradish peroxidase (Ni-HRP) (Mandel Scientific, Guelph, Ontario, Canada) as previously described (99). Plates were developed with a 3,3′,5,5′-tetramethylbenzidine (TMB) substrate system (Mandel Scientific) and read at an optical density at 600 nm (OD_600_) on a BioTek Synergy HT (Biotek, Nepean, Ontario, Canada). For data analysis, the OD_600_ of endothelial cells with no protein added was subtracted from OD_600_ values of endothelial cells incubated with recombinant protein. For FITC-Tp0751 binding assays, endothelial cells were preincubated with unlabeled Tp0751 (E115−P237) or Tp0327 (I23−S172) for 90 min at 37°C in an atmosphere of 5% CO_2_ as described above. Endothelial monolayers were washed three times with warm HBSS and incubated with 20 μM FITC-Tp0751 per well for 60 min at 37°C in an atmosphere of 5% CO_2_. Monolayers were washed three times with warm HBSS and binding of FITC-Tp0751 was evaluated by measuring fluorescence (485-nm excitation/528-nm emission). For data analysis, the fluorescence of wells containing endothelial cells with no protein added (FITC labeled or unlabeled) was subtracted from fluorescence values of all experimental wells. Data were normalized to wells of endothelial cells that had been incubated with FITC-Tp0751 but no unlabeled recombinant Tp0751 or Tp0327 (set at 100% and displayed as percent binding relative to the value for the FITC-Tp0751 control).

### Microscopy and cell counting.

Unless otherwise indicated, microscopy images were captured with a Cytation 5 Imaging Reader (BioTek) with a 20× objective at five random fields of view per well. For T. pallidum adhesion assays, samples were examined in a blind manner, and the number of endothelial nuclei (DAPI; 10 to 60 μm) and the number of adherent T. pallidum (FlaA; 2 to 8 μm) were measured from each field of view with the Cytation 5 software using size exclusion quantification settings (Biotek). To quantify adherent T. pallidum, the size threshold was set at a minimum of 2 μm and maximum of 8 μm, thereby excluding quantification of background fluorescence from endothelial cells. Endothelial cell quantification was achieved by counting nuclei using a size threshold ranging from 10 to 60 μm. The average count of adherent T. pallidum was normalized to the number of endothelial cells per field of view.

### Immunofluorescence-based T. pallidum-endothelial attachment assays.

Eight-well chamber slides were coated with phenol red-free Matrigel (Corning, Tewsbury, MA) by incubating with 500 μg/ml Matrigel for 1 h at RT. Equal densities of HUVECs or hCMEC/d3 cells were seeded into Matrigel-coated eight-well chamber slides (LabTek) and grown for 48 h at 37°C in 5% CO_2_ to form confluent monolayers. T. pallidum was isolated from rabbit testicular extractions and quantified using a Petroff-Hausser counting chamber (Hausser Scientific, Horsham, PA), and 5 × 10^6^ T. pallidum bacteria were added to each well of endothelial cells. All T. pallidum incubations were performed at 34°C in 1.5 to 5% O_2_ with 5% CO_2_ balanced with N_2_. For Tp0751 serum inhibition assays, T. pallidum isolated from testicular extractions was incubated with a 1:2 dilution of heat-inactivated anti-Tp0751 or anti-Tp0327 serum for 90 min, then added to HBSS-washed endothelial monolayers, and incubated for 60 min. Each well was washed four times with 10% NRS in 0.9% NaCl (warmed to 34°C), fixed in 10% formalin, and permeabilized with 0.05% Triton X-100 (TX-100). Endothelial attachment of T. pallidum was assessed by quantifying adherent cells using immunofluorescence detection of the major flagellar protein FlaA. Monolayers were blocked with 4% BSA in PBS and incubated with 1/1,000 dilution of chicken anti-T. pallidum FlaA serum (43) (preabsorbed with testicular extract and NRS) and 0.5 μg/ml mouse anti-human VE-cadherin (R&D Systems, Minneapolis, MN) for 1 h at RT, washed three times in PBS, and incubated with 1/1,000 goat anti-chicken A568 (ThermoFisher Scientific Inc., Burnaby, British Columbia, Canada) and 1/1,000 goat anti-mouse A488 (ThermoFisher Scientific) for 1 h at RT. Monolayers were counterstained with 3 μM DAPI (4′,6-diamidino-2-phenylindole; SigmaAldrich) for 5 min at RT, washed with PBS, and mounted with Vectashield mounting medium (Vector Laboratories, Brockville, Ontario, Canada). Secondary antibody controls were performed to confirm the specificity of the antibody staining (see Fig. S2 in the supplemental material). Viability and outer membrane integrity of T. pallidum were confirmed by sampling from each well prior to fixation. Viability was evaluated by visualizing the motility of treponemal samples on glass slides (ThermoFisher Scientific) on a Nikon Eclipse E600 dark-field microscope (Nikon Canada, Mississauga, Ontario, Canada) with a 40× objective (×400 magnification). Membrane integrity of T. pallidum was assessed by fixing samples onto CC^2^ eight-well chamber slides (ThermoFisher Scientific) using 2% PFA. Paired samples were either left unpermeabilized or permeabilized with 0.05% TX-100 and then were analyzed by epiimmunofluorescence detection of FlaA (processed as described above) on a Nikon 80i fluorescence microscope (Meridian Instrument Company, Inc., Kent, WA) fitted with a monochrome digital camera, a dark-field condenser, and fluorescein, DAPI, and Texas Red filters. Images were acquired with a 100× oil immersion objective (×1,000). For LamR inhibition assays, hCMEC/d3 monolayers were preincubated with polyclonal antibodies raised against residues 263–282 of LamR (51) or GAPDH antibodies for 1 h prior to the addition of T. pallidum.

### Quantitative real-time PCR-based T. pallidum-endothelial attachment assays.

Twenty-four-well tissue culture plates (Corning) were coated with Matrigel and equal densities of HUVECs were seeded to form confluent monolayers after 48 h at 37°C in 5% CO_2_. T. pallidum was isolated from rabbit testicular extractions and quantified using a Petroff-Hausser counting chamber (Hausser Scientific, Horsham, PA), and 2 × 10^6^ T. pallidum bacteria were added to each well of endothelial cells. All T. pallidum incubations were performed at 34°C in 1.5 to 5% O_2_ with 5% CO_2_ balanced with N_2_. For Tp0751 serum inhibition assays, T. pallidum isolated from testicular extractions was incubated with a 1:2 dilution of heat-inactivated antiserum for 60 min, then added to HBSS-washed endothelial monolayers, and incubated for 60 min. Each well was washed four times with 10% NRS in 0.9% NaCl (warmed to 34°C under microaerophilic conditions), then lysed in 10 mM Tris (pH 8.0), 0.1 M EDTA, and 0.05% SDS. Lysed samples were digested with proteinase K (Qiagen, Valencia, CA, USA) for 10 min at 56°C, followed by proteinase K heat inactivation for 10 min at 70°C before flash freezing in liquid nitrogen.

### DNA extraction and quantitative real-time PCR.

Extraction of T. pallidum and HUVEC genomic DNA (gDNA) was performed with a DNEasy blood and tissue extraction spin column kit (Qiagen) per the manufacturer’s instructions. Samples were eluted in 30 μl RNase-free DNase-free PCR grade distilled water (ThermoFisher Scientific), the DNA concentration was determined (Beckman Coulter DU 730 spectrophotometer, Mississauga, Ontario, Canada), and quantitative real-time PCR (qPCR) was performed on gDNA extractions of the T. pallidum-endothelial adhesion assays. Quantification of T. pallidum gDNA was determined using high-performance liquid chromatography (HPLC)-purified primers (Integrated DNA Technologies, Coralville, IA) targeting the endoflagellar sheath protein (*flaA*) gene (GenBank accession number M63142.1). The sense primer (5′-AACGCAAACGCAATGATAAA-3′) anneals to bases 475 to 494, and the antisense primer (5′-CCAGGAGTCGAACAGGAGATAC-3′) anneals to bases 738 to 759 of *flaA*. A *flaA* standard curve was generated using a 10-fold serial dilution from 10^7^ to 10^1^ copies of linearized plasmid DNA with an efficiency of 90.8% and an *R*^2^ value of 0.997 (49). Quantification of HUVEC gDNA was determined using HPLC-purified primers (Integrated DNA Technologies) targeting exon 6 of the glyceraldehyde-3-phosphate dehydrogenase (*GAPDH*) gene (GenBank accession number NG_007073). The sense primer (5′-AGCAAGAGCACAAGAGGAAGAG-3′) anneals to bases 3676 to 3697, and the antisense primer (5′-GAGCACAGGGTACTTTATTGATGG-3′) anneals to bases 3827 to 3850. A *GAPDH* standard curve was generated using a twofold serial dilution of HUVEC gDNA from 84.1 μg/μl to 0.041 ng/μl with an efficiency of 98.4% and an *R*^2^ value of 0.981. All reactions (20 μl) were performed in triplicate with SsoFast Evagreen Supermix (Bio-Rad, Mississauga, Ontario, Canada), 500 nM *flaA* or *GAPDH* primers, and 5 μl of template. All samples were diluted to the lowest DNA concentration, and *flaA* quantitation was normalized to picograms of GAPDH. Assays were run on a Bio-Rad CFX Connect Real-Time PCR Detection System (Bio-Rad, Mississauga, Ontario, Canada) using twin.tec white skirted 96-well plates (Eppendorf, Mississauga, Ontario, Canada) sealed with microseal B film (Bio-Rad). PCR conditions for *flaA* were as follows: (i) 95°C for 2 min; (ii) 40 cycles, with 1 cycle consisting of 95°C for 10 s and 65°C for 15 s. Following 40 cycles, there was a 95°C melt for 10 s, and then the melt curve was measured from 65°C to 95°C in 0.5°C increments for 5 s each. PCR conditions for *GAPDH* were as follows: (i) 95°C for 2 min; (ii) 40 cycles, with 1 cycle consisting of 95°C for 10 s and 60°C for 15 s. Following 40 cycles, there was a 95°C melt for 10 s, and then the melt curve was measured from 60°C to 95°C in 0.5°C increments for 5 s each. Each assay was run with the following controls: no-template control, no-primer control, and a positive control, including *flaA* plasmid DNA as the template. Data were analyzed using Bio-Rad Maestro software version 4.1.2.

### Endothelial cell receptor identification.

Affinity chromatography and mass spectrometry were performed as previously described (97), with select modifications. Two separate experiments were performed using different endothelial cell lines, recombinant Tp0751 constructs, and mass spectrometry sample preparation methods (Fig. S4; differences denoted in blue or green). Endothelial membrane- and membrane-associated proteins were isolated from HUVECs or hCMEC/d3 cell monolayers using a ThermoFisher Scientific Mem-PER eukaryotic membrane protein extraction kit per the manufacturer’s instructions (ThermoFisher Scientific). HUVEC proteins were isolated from 3.5 × 10^7^ cells after endothelial cells were lifted with trypsin and scraping, while hCMEC/d3 cells were physically lifted by scraping. The endothelial “prey” samples were immediately exposed to protease inhibitor cocktail V (Millipore) and dialyzed three times against 150-fold volumes of Tris-buffered saline (TBS) supplemented with 0.05% CHAPS [(3-((3-chloamidopropyl) dimethylammonio-1-propanesulfonate)] at 4°C using Slide-a-Lyzer dialysis cassettes (ThermoFisher), followed by the addition of CHAPs and imidazole to a final concentration of 0.5% and 40 mM, respectively. Isolation of Tp0751-interacting endothelial proteins was achieved using a ProFound Pull-Down PolyHis Protein:Protein Interaction kit per the manufacturer’s instructions (ThermoFisher Scientific). The “bait” proteins, recombinant Tp0751 (C24−P237) or Tp0751 (E115−P237), were dialyzed against TBS−0.05% CHAPS buffer at 4°C, followed by the addition of CHAPS to a final concentration of 0.5%, and 150 μg of recombinant Tp0751 was added to the immobilized cobalt chelate and incubated for 2 h at 4°C. Immobilized Tp0751 was washed three times with TBS−0.5% CHAPS prior to the addition of 500 μl of enriched membrane protein from endothelial cells. The bait-prey samples were coincubated for 16 h at 4°C and washed three times with 50% TBS in ProFound lysis buffer with 10 mM imidazole. In parallel, bait-only and prey-only columns were processed. Per the manufacturer’s instructions, Tp0751-interacting prey proteins were captured by incubating the samples with 50% TBS in ProFound lysis buffer with 290 mM imidazole for 10 min at 4°C. Samples were subjected to in-gel or in-solution tryptic digestion before analysis by liquid chromatography tandem mass spectrometry on a LTQ Velos Orbitrap with Easy nLC-II (ThermoScientific). Data analysis was performed using Mascot (Matrix Science, Boston, MA) or Scaffold (Proteome Software, Portland, OR), and candidate endothelial receptors for Tp0751 were identified based upon peptides that achieved a significant mascot score or scaffold probability, were found exclusively in the bait-prey sample in both experiments, had greater than one unique significant peptide hit, and were determined through literature analyses to localize to the surfaces of host cells.

### Immunofluorescence detection of LamR.

Surface-localized LamR was detected from nonpermeabilized endothelial monolayers fixed with 4% PFA using mouse anti-LamR (Novus Biologicals, Littleton, CO) and goat anti-mouse A568 (ThermoFisher). Validation of intact endothelial membranes was achieved with a control immunofluorescence experiment to detect internal protein GAPDH in permeabilized and nonpermeabilized endothelial cells using a mouse anti-GAPDH primary antibody (ThermoFisher Scientific) and a secondary goat anti-mouse Alexa Fluor 488 (ThermoFisher Scientific).

### Coimmunoprecipitation.

Live hCMEC/d3 monolayers were coincubated with 25 μM recombinant Tp0751 (E115−P237) for 60 min at 37°C and 5% CO_2_. Monolayers were washed with HBSS, and lysates were harvested with M-PER mammalian protein extract reagent (ThermoFisher Scientific) in the presence of the protease inhibitor cocktail set V (Calbiochem, San Diego, CA), incubated on ice for 20 min, and centrifuged at 20,000 × *g* for 15 min at 4°C. Protein concentration was determined with a bicinchoninic acid (BCA) assay (ThermoFisher Scientific). Mouse monoclonal anti-LamR antibodies (MLuc5; ThermoFisher Scientific) were covalently coupled to protein G magnetic beads (Dynabeads; ThermoFisher Scientific) using bis(sulfosuccinimidyl)suberate (BS^3^; ThermoFisher Scientific) and coincubated with 75 μg of hCMEC/d3 lysate for 2 h at 4°C. Beads were washed with M-PER lysis buffer and then resuspended in sodium dodecyl sulfate-polyacrylamide gel electrophoresis (SDS-PAGE) loading buffer containing dithiothreitol (DTT) (ThermoFisher) and heated to 95°C to elute interacting proteins. Protein interactions were analyzed by SDS-PAGE and immunoblotting with rabbit anti-LamR (R&D Biosystems) and goat anti-rabbit IRdye680 secondary antibodies (Li-Cor, Lincoln, NE) to detect LamR and Tp0751-specific mouse monoclonal antibodies (Immunoprecise Antibodies, Victoria, British Columbia, Canada) with goat anti-mouse IRdye800 secondary antibodies (Li-Cor) to detect Tp0751. Lysates were also subject to quantitative immunoblotting using rabbit anti-LamR (R&D Biosystems) and goat anti-rabbit IRdye680 secondary antibodies (Li-Cor) to detect LamR and mouse anti-GAPDH (R&D Biosystems) and goat anti-mouse IRdye800 secondary antibodies (Li-Cor) to detect GAPDH. LamR band intensity was normalized to band intensity of the internal loading control GAPDH. All immunoblots were visualized on an Odyssey CLx imaging system (Li-Cor), and fold enrichment of LamR was determined by measuring band intensity using the Li-Cor Image Studio software.

### Plate-based binding assays.

Maxisorp 384-well plates (ThermoFisher Scientific) were coated with 0.1 μg/well of recombinant Tp0751 (V99–P237) or Tp0327 (I23–S172). Following a blocking step, 0.5 μM commercially purchased recombinant LamR (Novus Biologicals, Littleton, CO) was added to each well. LamR binding to Tp0751 or Tp0327 was evaluated using a rabbit polyclonal anti-LamR antibody (1/500; Genscript, Piscataway, NJ), and an HRP-conjugated goat anti-rabbit IgG (1/10,000; SigmaAldrich). In a reciprocal binding assay, plates were coated with 0.1 μg/well of commercially purchased recombinant LamR (Novus Biologicals) and 2 μM His_6_ recombinant Tp0751 (V99–P237) or Tp0327 (I23–S172) was added to each well. In addition, His_6_ Tp0751 synthetic peptides p1 (amino acids 46 to 69 [aa 46–69]), p4 (aa 88–112), p6 (aa 117–141), p9 (aa 158–181), p10 (aa 172–195), and p11 (aa 185–208) (42) were added to each LamR-coated well. Binding of treponemal proteins or synthetic Tp0751 peptides was evaluated using Ni-HRP (Mandel Scientific) as previously described (44, 45). In all binding assays, coated wells were blocked with 1% BSA in Tris-buffered saline with 0.1% Tween 20 (TBS-T), and all washes were performed with TBS-T. Binding assays were developed with a TMB substrate system (Mandel Scientific) and read at OD_600_ on a BioTek Synergy HT. For data analysis, the OD_600_ values from wells coated with LamR or recombinant treponemal protein were subtracted from OD_600_ values of all experimental wells.

### Statistical analyses.

Statistical analyses were performed in GraphPad Prism (GraphPad, La Jolla, CA) as denoted in figure legends. Graphs were prepared in GraphPad Prism (GraphPad) or Excel (Microsoft, Redmond, WA).
