# Supplementary figures and images for: Identification of the Neuroinvasive Pathogen Host Target, LamR, as an Endothelial Receptor for the Treponema pallidum Adhesin Tp0751
Source: mSphere. 2020 Apr 1;5(2):e00195-20. doi: 10.1128/mSphere.00195-20 (PMC7113585; doi:10.1128/mSphere.00195-20)

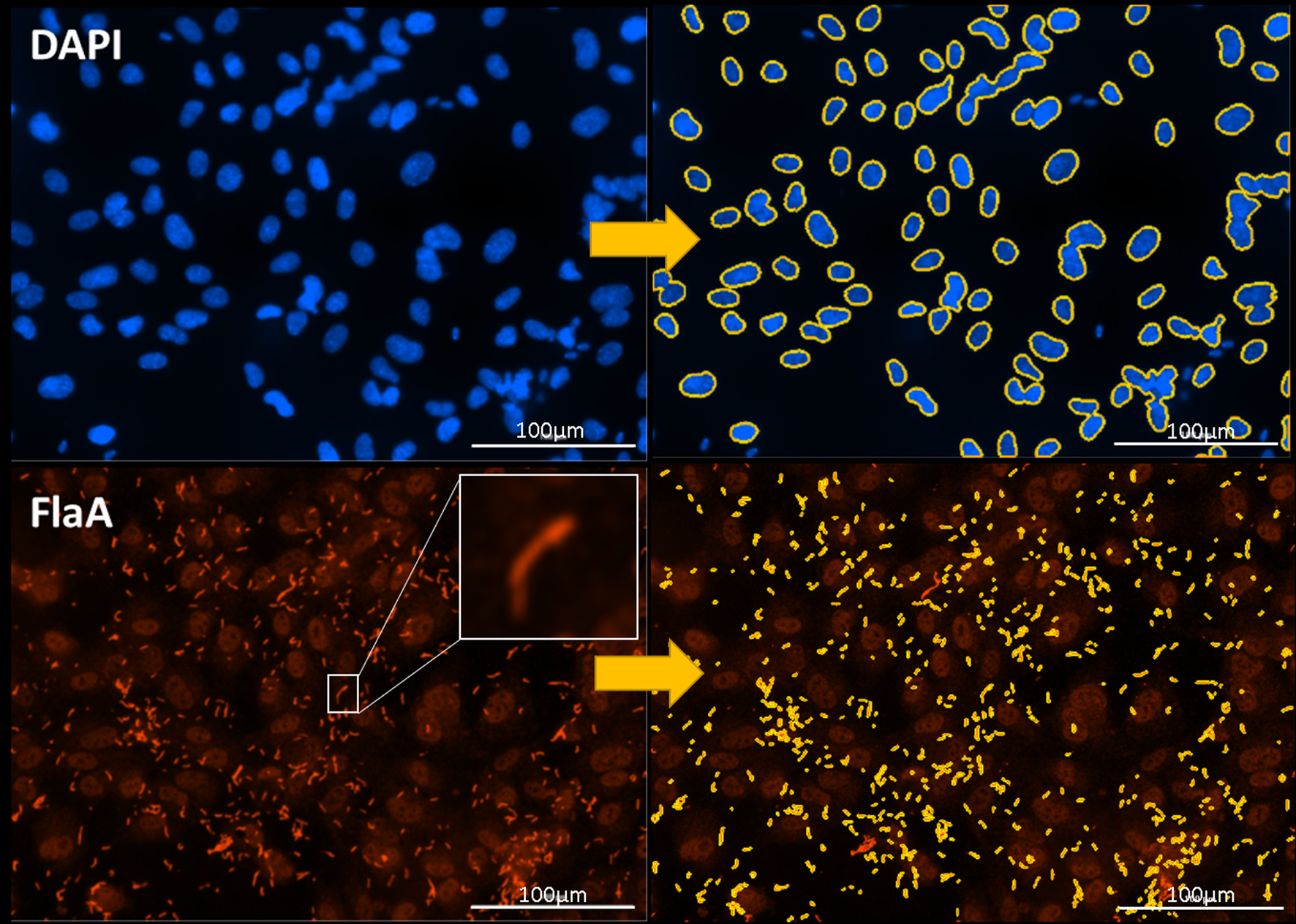

Supplement: FIG S1 [file mSphere.00195-20-sf001.tif]

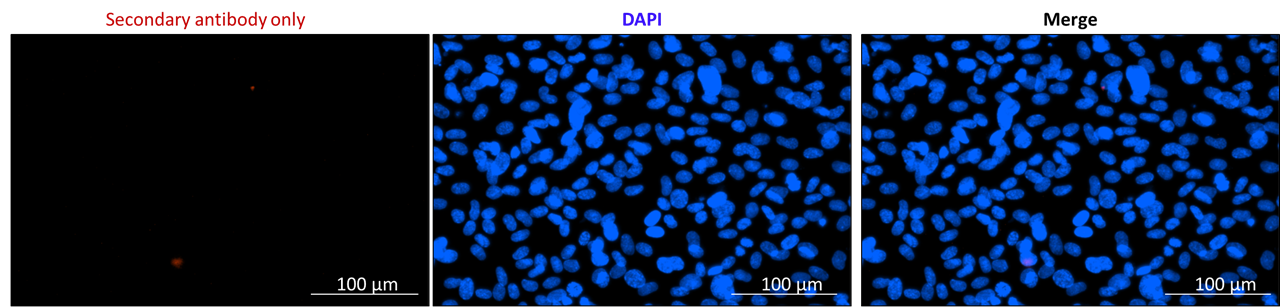

Supplement: FIG S2 [file mSphere.00195-20-sf002.tif]

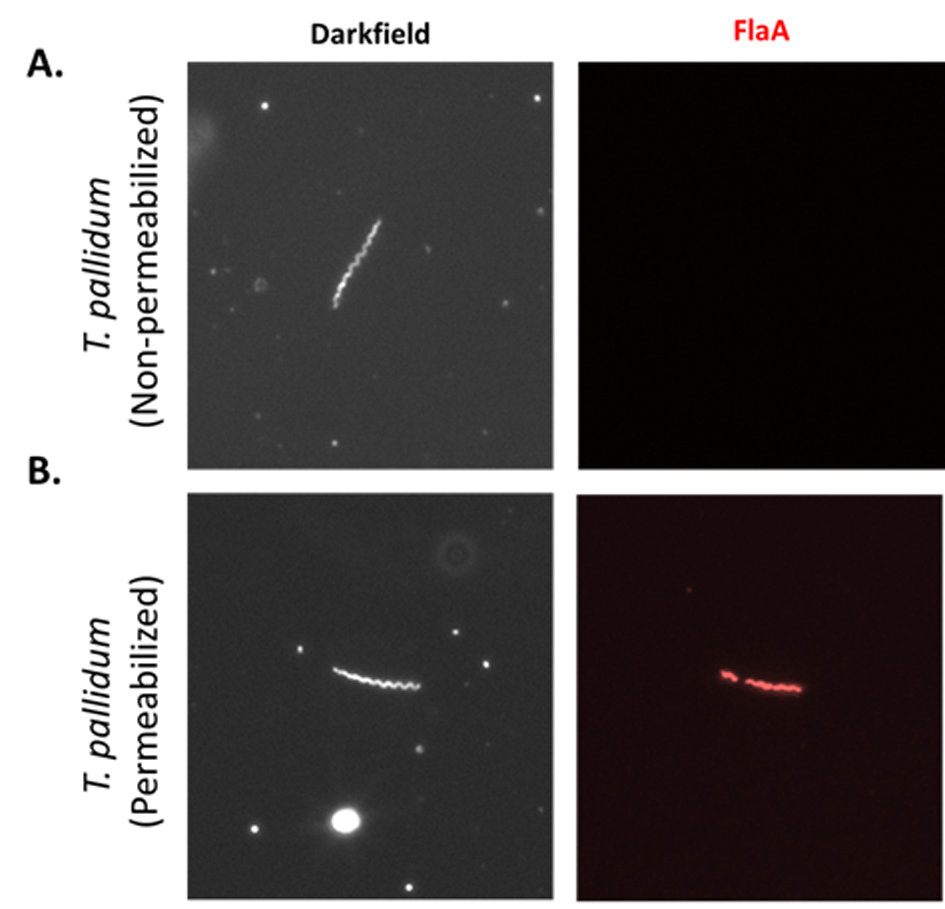

Supplement: FIG S3 [file mSphere.00195-20-sf003.tif]

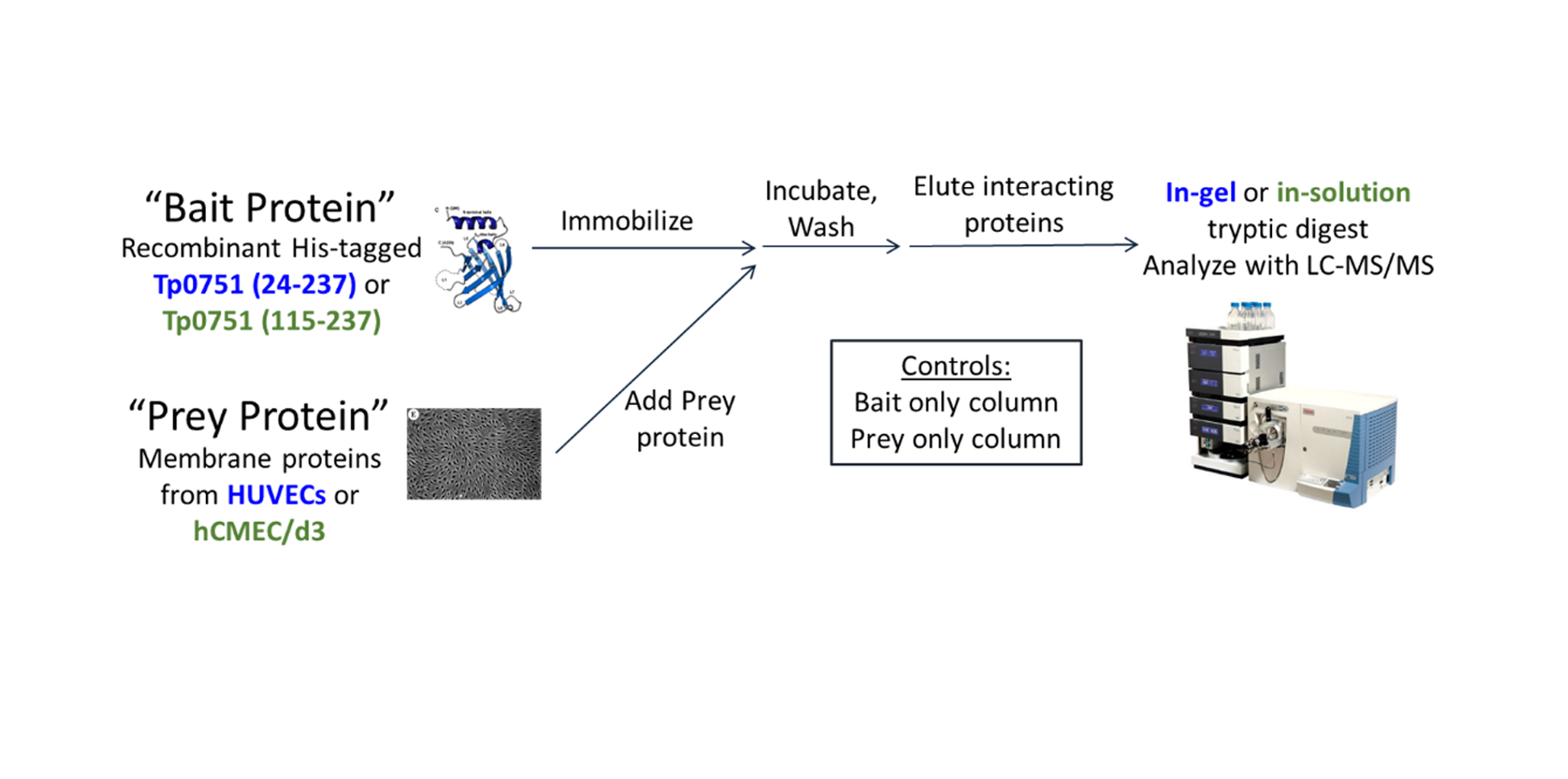

Supplement: FIG S4 [file mSphere.00195-20-sf004.tif]

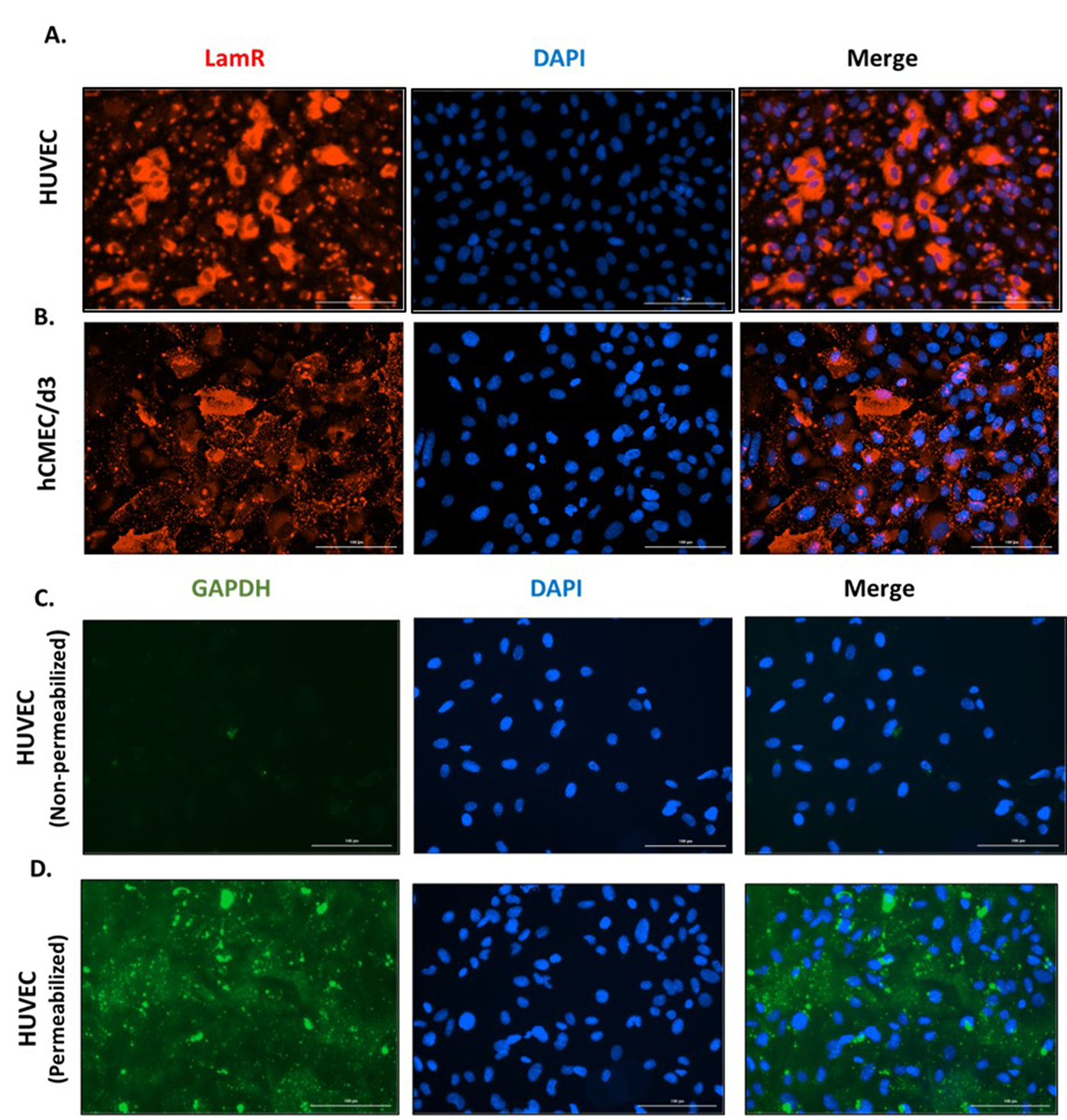

Supplement: FIG S5 [file mSphere.00195-20-sf005.tif]
