# Supplementary material for: Identification of the Neuroinvasive Pathogen Host Target, LamR, as an Endothelial Receptor for the Treponema pallidum Adhesin Tp0751
Source: mSphere. 2020 Apr 1;5(2):e00195-20. doi: 10.1128/mSphere.00195-20 (PMC7113585; doi:10.1128/mSphere.00195-20)
Supplement: TABLE S1 [file mSphere.00195-20-st001.docx]

| **hCMEC/d3 Protein** | **Uniprot Accession Number** | **Scaffold Probability*^a^*** | **# of Peptides*^b^*** | **Peptide Sequences** | **Total Sequence Coverage*^c^*** | **Predicted localization** |
| --- | --- | --- | --- | --- | --- | --- |
| 67 kDa laminin receptor | P08865 | 98% | 4 | (K)FLAAGTHLGGTNLDFQMEQYIYK(R) | 23% | Cell surface, cytoplasm, nucleus |
|  |  |  |  | (R)AIVAIENPADVSVISSR(N) |  |  |
|  |  |  |  | (K)FAAATGATPIAGR(F) |  |  |
|  |  |  |  | (R)FTPGTFTNQIQAAFR(E) |  |  |
| Stomatin | P27105 | 98% | 2 | (K)EASMVITESPAALQLR(Y) | 15% | Cell surface (lipid rafts) |
|  |  |  |  | (R)VQNATLAVANITNADSATR(L) |  |  |
| *^a^*Only proteins identified with a Scaffold probability ≥95 % were considered potentially significant. *^b^*No. of observed peptides include all peptides that differ only by sequence and that were identified with a Scaffold probability ≥95 %; peptides with the same sequence but modification or charge differences are not included. Proteins that were identified with a Scaffold probability ≥95 % but with only one observed peptide were not considered.  *^c^*Total Sequence Coverage is based on all identified peptides with unique sequences | | | | | | |

**Table S1. Tp0751 (E115-P237)-interacting hcMEC/d3 integral membrane and membrane-associated proteins**

**identified by affinity chromatography and mass spectrometry.**

|  |
| --- |
