# Supplementary material for: Identification of the Neuroinvasive Pathogen Host Target, LamR, as an Endothelial Receptor for the Treponema pallidum Adhesin Tp0751
Source: mSphere. 2020 Apr 1;5(2):e00195-20. doi: 10.1128/mSphere.00195-20 (PMC7113585; doi:10.1128/mSphere.00195-20)
Supplement: TABLE S2 [file mSphere.00195-20-st002.docx]

**Table S2. Tp0751 (C24-P237)-reactive HUVEC integral membrane and membrane associated proteins identified by affinity chromatography and mass spectrometry**

| **HUVEC Protein** | **Uniprot Accession Number** | **Mascot Score*^a^*** | **# of Peptides*^b^*** | **Peptide Sequences** | **Total Sequence Coverage*^c^*** | **Predicted localization** |
| --- | --- | --- | --- | --- | --- | --- |
| 67 kDa laminin receptor | P08865 | 78 | 4 | (K)SDGIYIINLK(R) | 23% | Cell surface, cytoplasm, nucleus |
|  |  |  |  | (R)FTPGTFTNQIQAAFR(E) |  |  |
|  |  |  |  | (R)AIVAIENPADVSVISSR(N) |  |  |
|  |  |  |  | (R)ADHQPLTEASYVNLPTIALCNTDSPLR(Y) |  |  |
| Stomatin | P27105 | 157 | 5 | (R)ILQGGAKGPGLFFILPCTDSFIKVDMR(T) | 35% | Cell surface (lipid rafts) |
|  |  |  |  | (R)ALKEASMVITESPAALQLR(Y) |  |  |
|  |  |  |  | (K)EASMVITESPAALQLR(Y) |  |  |
|  |  |  |  | (K)NSTIVFPLPIDMLQGIIGAK(H) |  |  |
|  |  |  |  | (R)VQNATLAVANITNADSATR(L) |  |  |
| ***^a^***Only proteins identified with a significant Mascot score were considered potentially significant. ***^b^***No. of observed peptides include all peptides that differ only by sequence and that were identified with a significant Mascot score; peptides with the same sequence but modification or charge differences are not included. Proteins that were identified with a significant Mascot score but with only one observed peptide were not considered. ***^c^***Total Sequence Coverage is based on all identified peptides with unique sequences. | | | | | | |
|  | | | | | |  |
